# Biophysical and Biochemical Comparison of Extracellular Vesicles Produced by Infective and Non-Infective Stages of *Trypanosoma cruzi*

**DOI:** 10.3390/ijms22105183

**Published:** 2021-05-13

**Authors:** Lissette Retana Moreira, Alexa Prescilla-Ledezma, Alberto Cornet-Gomez, Fátima Linares, Ana Belén Jódar-Reyes, Jorge Fernandez, Ana Karina Ibarrola Vannucci, Luis Miguel De Pablos, Antonio Osuna

**Affiliations:** 1 Grupo de Bioquímica y Parasitología Molecular (CTS 183), Departamento de Parasitología, Campus de Fuentenueva, Instituto de Biotecnología, Universidad de Granada, 18071 Granada, Spain; lissette.retanamoreira@ucr.ac.cr (L.R.M.); alexap@correo.ugr.es (A.P.-L.); acornetgomez@ugr.es (A.C.-G.); ana_karinai@hotmail.com (A.K.I.V.); 2 Departamento de Parasitología, Facultad de Microbiología, Universidad de Costa Rica, San José 2060, Costa Rica; 3 Centro de Investigación en Enfermedades Tropicales (CIET), Universidad de Costa Rica, San José 2060, Costa Rica; 4 Departamento de Microbiología Humana, Facultad de Medicina, Universidad de Panamá, Ciudad de Panamá 3366, Panama; 5 Unidad de Microscopía de Fuerza Atómica, Centro de Instrumentación Científica, Universidad de Granada, 18003 Granada, Spain; flinaor@ugr.es; 6 Grupo de Física de Fluidos y Biocoloides (FQM 115), Excellence Research Unit Modeling Nature (MNat), Departamento de Física Aplicada, Facultad de Ciencias, Universidad de Granada, 18071 Granada, Spain; ajodar@ugr.es; 7 Departamento de Química Analítica, Facultad de Ciencias, Universidad de Granada, 18071 Granada, Spain; jffernan@ugr.es; 8 Unidad de Proyectos, Convenios e Investigación, SENEPA, Ministerio de Salud, 001221 Asunción, Paraguay; 9 Departamento de Biotecnología, Universidad Nacional de Asunción, 1039 Asunción, Paraguay

**Keywords:** extracellular vesicles, *Trypanosoma cruzi*, epimastigote, trypomastigote, proteins, *trans*-sialidase, mechanical properties, atomic force microscopy, zeta-potential

## Abstract

Extracellular vesicles (EVs) are small lipid vesicles released by either any prokaryotic or eukaryotic cell, or both, with a biological role in cell-to-cell communication. In this work, we characterize the proteomes and nanomechanical properties of EVs released by tissue-culture cell-derived trypomastigotes (mammalian infective stage; (TCT)) and epimastigotes (insect stage; (E)) of *Trypanosoma cruzi*, the etiologic agent of Chagas disease. EVs of each stage were isolated by differential centrifugation and analyzed using liquid chromatography with tandem mass spectrometry (LC-MS/MS), dynamic light scattering (DLS), nanoparticle tracking analysis (NTA), electron microscopy and atomic force microscopy (AFM). Measurements of zeta-potential were also included. Results show marked differences in the surface molecular cargos of EVs between both stages, with a noteworthy expansion of all groups of *trans*-sialidase proteins in trypomastigote’s EVs. In contrast, chromosomal locations of *trans*-sialidases of EVs of epimastigotes were dramatically reduced and restricted to subtelomeric regions, indicating a possible regulatable expression of these proteins between both stages of the parasite. Regarding mechanical properties, EVs of trypomastigotes showed higher adhesion compared to the EVs of epimastigotes. These findings demonstrate the remarkable surface remodeling throughout the life cycle of *T. cruzi*, which shapes the physicochemical composition of the extracellular vesicles and could have an impact in the ability of these vesicles to participate in cell communication in completely different niches of infection.

## 1. Introduction

Extracellular vesicles (EVs) are a diverse group of nanovesicles constitutively released by either any prokaryotic or eukaryotic cell, or both. These vesicles are involved in intercellular communication and include EVs and ectosomes, among others. EVs are a type of spherical or cup-shaped EV surrounded by a phospholipid bilayer of 30–150 nm in diameter [1,2] and originated in multivesicular bodies (MVB) of endocytic origin. Ectosomes are more heterogeneous in shape, larger in diametrical size (0.1–1 µm) and shed directly from the plasma membrane [3]. Their packed and released cargos are very diverse and include lipids, proteins, small metabolites and different populations of RNAs, dsDNA or ssDNA. This composition could vary according to different physiological statuses of the producer cell, such as cancer/non-cancerous cells or infected/non-infected cells by microorganisms [4,5,6,7].

Some parasites secrete EVs to empower their infectivity due to their ability to induce physiological changes in the cells with which they interact [8] and create an optimal niche at cellular and tissue levels [7,9,10]. *Trypanosoma cruzi*, the etiologic agent of Chagas disease, is a kinetoplastid protozoan parasite that affects approximately 8 million people globally, with some 300,000 new cases and 15,000 deaths annually [11]. The life cycle of the parasite encompasses a series of developmental transformations, including epimastigote forms (non-infective and replicative stage; (E)) in the digestive tract of the infected reduviid bug vector, and bloodstream trypomastigote forms (infective for cells and non-replicative) in the mammalian hosts. The secretion of EVs by this parasite was first reported by da Silveira et al. in 1979 [12].

There are several biochemical differences between trypomastigotes and epimastigotes, such as metabolism [13], lipid and protein composition of cell membranes [14,15] and its ability for division. Other differences include morphological changes such as the posterior or anterior positioning of the flagellum and kinetoplast, as well as the expansion of surface membrane protein glycocalyx of the trypomastigotes compared to epimastigotes. In this sense, MASPs, mucins, gp63 and *trans*-sialidase (TS) family proteins are over-represented in proteomic, glycoproteomic and RNA expression analysis of trypomastigotes [16,17,18]. Indeed, recent findings have described the release via EVs of *trans*-sialidase proteins, which are virulence factors implicated in the transfer of sialic acid from host glycoproteins to parasite mucins [19,20] and involved in the mechanism of parasite-host cell cytoadherence and invasion [20,21,22,23]. Immunofluorescence analysis using mAb 3F6 against gp82 TS proteins showed the delivery of this type of protein in EVs to host cells, where they probably modulate cellular activity, increasing the parasite’s survival and replication [24]. Moreover, during the invasion process of *T. cruzi*, host cells increase their EV shedding in a calcium-dependent manner [8,25,26] and load parasite proteins (up to 25% for metacyclic trypomastigotes) in EVs, thus exporting proteins from intracellular stages of the parasite to the extracellular milieu that could be implicated in the pathological outcomes of the infection with *T. cruzi* [25].

In this work, we performed an extensive characterization and compared proteomes and biophysical properties of EVs produced by the E and tissue-culture cell-derived trypomastigotes (TCT) of *T. cruzi.* The data obtained reflects the drastic changes in the protein composition as well as the nanomechanical properties of the vesicles produced by both stages of the parasite, differences that could have clear implications in the parasite’s survival in two radically different biological niches.

## 2. Results

### 2.1. Purification and Characterization of EVs

**Figure 1:**
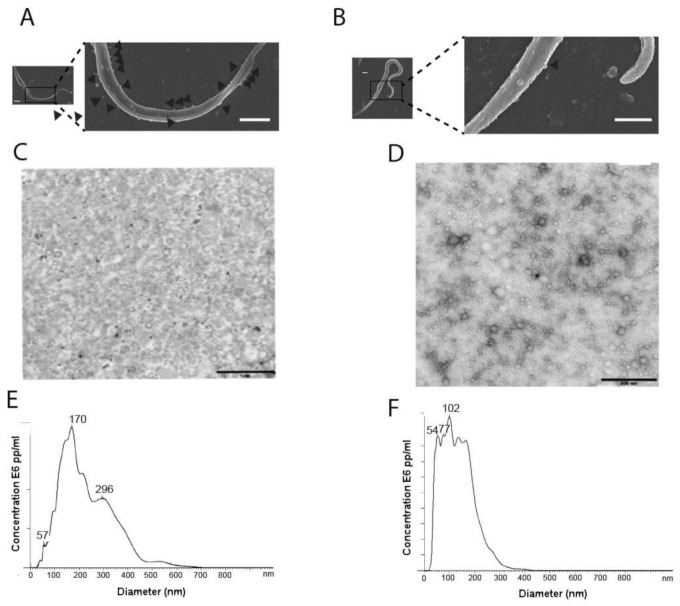
Purification of the extracellular vesicles (EVs) of epimastigotes (E) and EVs of tissue-culture cell-derived trypomastigotes (TCT) of *T. cruzi* (Pan4 strain, DTU I). Scanning electron microscopy (SEM) of E (**A**) and TCT (**B**) employed in this study (scale bar: 1 µm). (**C**) Transmission electron microscopy (TEM) of purified EVs of E (scale bar: 1 µm). (**D**) TEM of purified EVs of TCT (scale bar: 500 nm). (**E**) Nanoparticle tracking analysis (NTA) size distribution of purified EVs of E. (**F**) NTA size distribution of purified EVs of TCT. Representative figures and graphs of 7 different replicates are shown.

To obtain EVs of E and TCT, a procedure including differential centrifugation, coupled to a filtration process through 0.22 µm pore filters and ultracentrifugation was employed (Figure S1). After the isolation and purification process, transmission electron microscopy (TEM) analysis revealed the success of the methodology employed in separating the EVs (Figure 1). Regarding nanoparticle tracking analysis (NTA), the mean size of most of the vesicles secreted by E was 259 ± 21 nm and the mode was 1712 ± 11 nm, while the mean size of the vesicles of TCT was 143 ± 24 nm and the mode was 63 ± 24 nm (Figure 1E,F). These results were similar to measurements obtained by dynamic light scattering (DLS) in the case of EVs of E (mean size: 183 ± 22 nm); however, in the case of the EVs of TCT, the resulting mean size was smaller (60 ± 17 nm). Thus, it is shown that the EVs secreted by TCT seem to be smaller than the EVs of E but both coincide with the reported size of EVs (Figure 1).

### 2.2. Proteomic Profile of EVs of E and TCT

**Figure 2:**
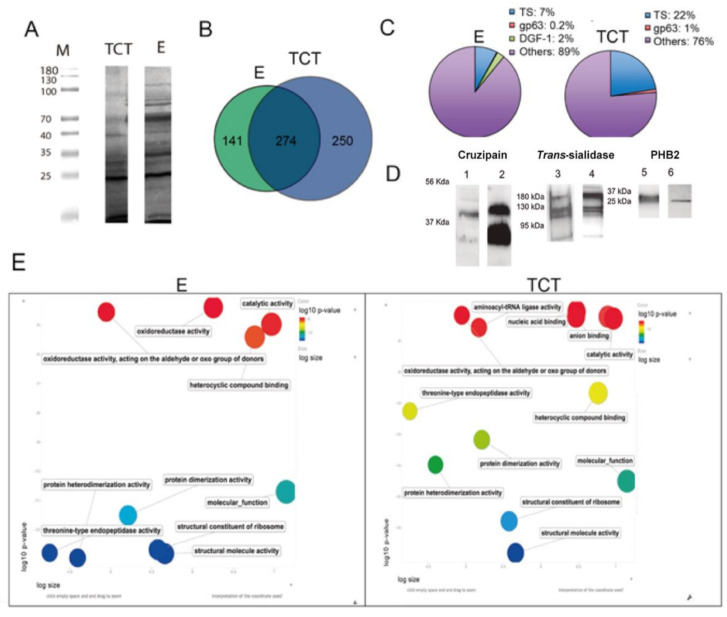
Qualitative proteomic analysis of the cargos of EVs of E and EVs of TCT of *T. cruzi*. (**A**) Western blot and differential protein profile of EVs of E and EVs of TCT using polyclonal anti-*T. cruzi* antibodies. (**B**) Venn diagram of specific and shared proteins of EVs of E and EVs of TCT. (**C**) Pie chart representing the percentage of proteins of EVs of E and EVs of TCT that belong to multigene families. (**D**) Western blot analysis for the confirmation of cruzipain, *trans*-sialidase [8] and prohibitin 2 (PHB2) in EVs of *T. cruzi:* (1) cruzipain in whole lysates of TCT; (2) cruzipain in EVs of TCT; (3) *trans*-sialidase (mAb 39) in whole lysates of TCT; (4) *trans*-sialidase (mAb 39) in EVs of TCT; (5) PHB2 in whole lysates of E; and (6) PHB2 in EVs of E. (**E**) Scatter plot representing Gene Ontology (GO) terms enrichment analysis of proteins of the EVs of E and EVs of TCT, categorized by molecular function (*p* ≤ 0.01).

Western blot analysis using polyclonal anti-*T. cruzi* antibodies revealed numerous proteins with different expression profiles, indicating the different nature of the protein cargos of EVs of E and EVs of TCT (Figure 2A). To generate an overview of the proteomic differences between the EVs of both stages, seven independent biological replicates (four for EVs of TCT and three for EVs of E) of 40 μg purified EVs were subjected to liquid chromatography with tandem mass spectrometry (LC-MS/MS). All proteins were searched against UniProt-*T. cruzi* CL Brener database and proteins present in at least two replicates, with two or more peptides identified, were used for further comparisons. As a result, 528 proteins were identified in the EVs of TCT and 415 proteins in the EVs of E (Table S1). In either EVs of E or EVs of TCT, the proteins found have different probabilities for appearance in a given sample (2/3 or 3/3 in E and 2/4, 3/4 and 4/4 in TCT). These results indicate that the protein cargos of EVs are either constitutively present or eventually exported through this route (Figure S2).

Of those proteins, 274 were present in the EVs of both stages, while 250 proteins (66%) were specific to EVs of TCT and 141 proteins (26.7%) were specific to EVs of E, revealing bigger expansion of TCT specific protein cargos (Figure 2B). These differences were mainly due to a differential and selective enrichment of surface proteins released by the TCT stage. Particularly, 23% of the proteome of EVs of TCT encompasses surface proteins belonging to multigene families, whereas only 9.2% of the proteome of EVs of E exhibits these kinds of proteins (Figure 2C). To confirm these results, antibodies against TS, cruzipain and PHB2 proteins were employed, and the presence of these proteins in EVs of TCT, and PHB2 protein in EVs of E, was detected (Figure 2D). The results also show stage-specific multigene surface protein cargos such as DGF-1 proteins, which were exclusively present in the EVs of E (2% of the protein content), and gp63 surface proteins only present in the EVs of TCT (1% of the protein content) (Figure 2C). In our analysis, we did not find proteins from other surface families such as mucins or MASPs.

In order to search for differential enzymatic activities, the proteomes of EVs of TCT and EVs of E were analyzed for differential Gene Ontology (GO) enrichments (*p* ≤ 0.01). As shown in Figure 2E, both proteomes showed similar patterns in many of the molecular activities contained with enrichments of threonine-type peptidase activity (GO:0070003), structural constituents of the ribosome (GO:0003735) and aminoacyl-tRNA ligase activity (GO:0004812). However, the analysis also showed a differential enrichment with 22 GO terms in EVs of TCT versus 12 for EVs of E (Table S2). Moreover, some enrichments such as purine ribonucleoside triphosphate binding (GO:0035639) or coenzyme binding (GO:0050662) were only significant in EVs of TCT.

Finally, the proteomic composition of EVs of TCT and EVs of E of the *T. cruzi* Pan4 strain employed in this study was compared to the previous proteome dataset from Bayer Santos et al. (2013), obtained from the *T. cruzi* DM28c strain (DTU I) [24]. The latter analyzed EV protein cargos in different vesicle fractions called V2, with a mean size of 131 nm (epimastigotes) and 143 nm (metacyclic trypomastigotes), and V16, with vesicles of 74 nm (epimastigotes) and 87 nm (metacyclic trypomastigotes). These fractions fall within the range of the exosome fractions of our study (see above). Surprisingly, only 26.7% of the protein content of EVs of E was shared between proteomes and 28% of the proteins of the EVs of metacyclic trypomastigotes were present in the EVs of TCT of the Pan4 strain (Figure S3).

### 2.3. Composition, Variability and Location of TS Proteins in EVs of E and TCT

**Figure 3:**
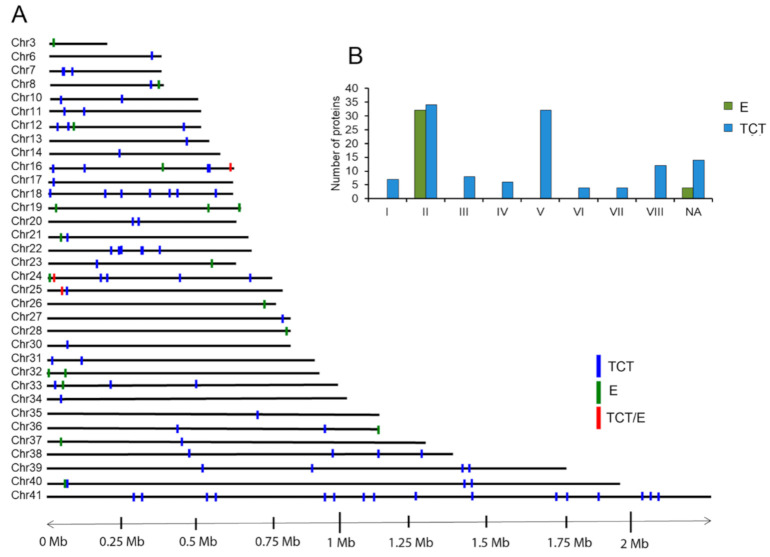
Mapping of TS proteins found in EVs of E and EVs of TCT of *T. cruzi* Pan4 strain on *T. cruzi* CL-Brener strain chromosomes. (**A**) Genomic mapping of TS on chromosomes represented as blue bars for TS of trypomastigote forms derived from cell cultures, green bars for TS of epimastigotes or red bars for TS genes present in both stages (TCT: *n* = 121; E: *n* = 36). (**B**) Bar graph representing the TS proteins found in the EVs of TCT and EVs of E and categorized for the CL-Brener strain in groups I–VII, according to Freitas et al. [27].

Among surface proteins, TS proteins showed the highest levels of protein diversity of EVs of TCT, comprising 22% of the total composition and 7% of the total cargo of EVs. To evaluate the TS protein composition, the proteins of the Pan4 strain identified in CL-Brener’s proteome were classified in the TS groups I–VIII proposed by Freitas et al. (2011) [7]. Extracellular vesicles of TCT showed high variability and co-expression of multiple TS (*n* = 121), with proteins falling within all TS groups (I–VIII), whereas the TS cargos in EVs of epimastigotes were far less abundant (*n* = 33) and restricted to group II (Figure 3).

TS were also mapped on *T. cruzi* chromosomes to determine whether or not there was an association between the TS expression and chromosomal location among EVs of E and EVs of TCT. As observed in the same figure, TSs of the EVs of TCT were randomly distributed all across *T. cruzi* chromosomes. In contrast and strikingly, TSs of EVs of E showed a clear bias towards both chromosomal ends (82%, 17/20 TS), indicating a restrictive expression pattern of the TS group II in epimastigotes.

It was also demonstrated that the TS protein cargos of EVs are also either constitutively present or eventually exported through the exosomal route in E and TCT, as shown in Figure S4.

**Figure 4:**
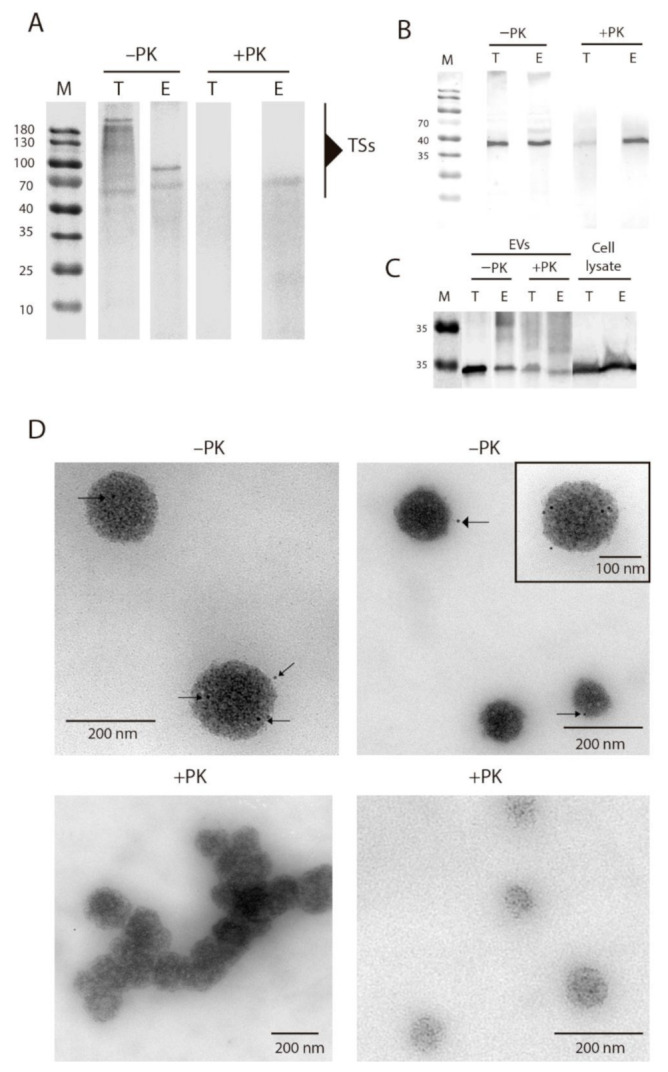
Location of TS proteins on the surface of EVs of *T. cruzi.* (**A**) Western blot of EVs of E and EVs of TCT treated/untreated (+PK/−PK, respectively) with proteinase K and incubated with anti-TS antibodies. All lanes were loaded with an equal amount of protein (30 µg). (**B**) Western blot of EVs of E and EVs of TCT treated/untreated (+PK/−PK, respectively) with proteinase K and incubated with anti-cruzipain antibodies. All lanes were loaded with an equal amount of protein (30 µg). (**C**) Western blot of EVs of E and EVs of TCT treated/untreated with proteinase K and whole lysates of TCT and E incubated with anti-calmodulin antibodies. All lanes were loaded with equal amount of protein (30 µg). (**D**) Immunogold labeling of TS proteins in EVs of TCT treated/untreated with proteinase K. Black arrows indicate the gold particles and thus TS location.

Finally, with the aim of eliminating proteins present at the surface of EVs and to ascertain whether or not TS proteins are membrane-bound cargos, EVs of E and EVs of TCT were treated with proteinase K and analyzed by Western blot and TEM, using immunogold labeling (Figure 4). While the untreated EVs of TCT (−PK) showed the characteristic TS pattern of high molecular weight proteins (Figure 4A), in the case of EVs treated with proteinase (+PK), the TS residues were practically removed, with only a minor band of approximately 65 kDa that also appears in the untreated EVs of E. This band could correspond to a protein or a domain of the TS possibly vehiculated inside the membranes of the EVs. Similarly, in the immunogold labelling, EVs untreated with proteinase K showed gold marks on their surface that were not present in EVs treated with the proteinase (Figure 4C). The presence of other parasite-specific proteins like cruzipain (gp57/51) was not affected by the treatment, which indicates that the enzyme could be located inside the vesicles, therefore being inaccessible to the treatment with proteinase K (Figure 4B).

Results obtained in these experiments confirm the presence of TS proteins on the surface of EVs, regardless of the group (I–VI) they belong to. This location of TS on the surface of EVs would be similar to what occurs on the surface of the parasite, where TSs are bound to the membrane [28]. Besides, it was also proved that the EV’s integrity is not compromised after the treatment with proteinase K.

### 2.4. Biophysical Properties of EVs of E and TCT

#### 2.4.1. Atomic Force Microscopy (AFM) Analysis

##### Topographic Measurements and Imaging

**Figure 5:**
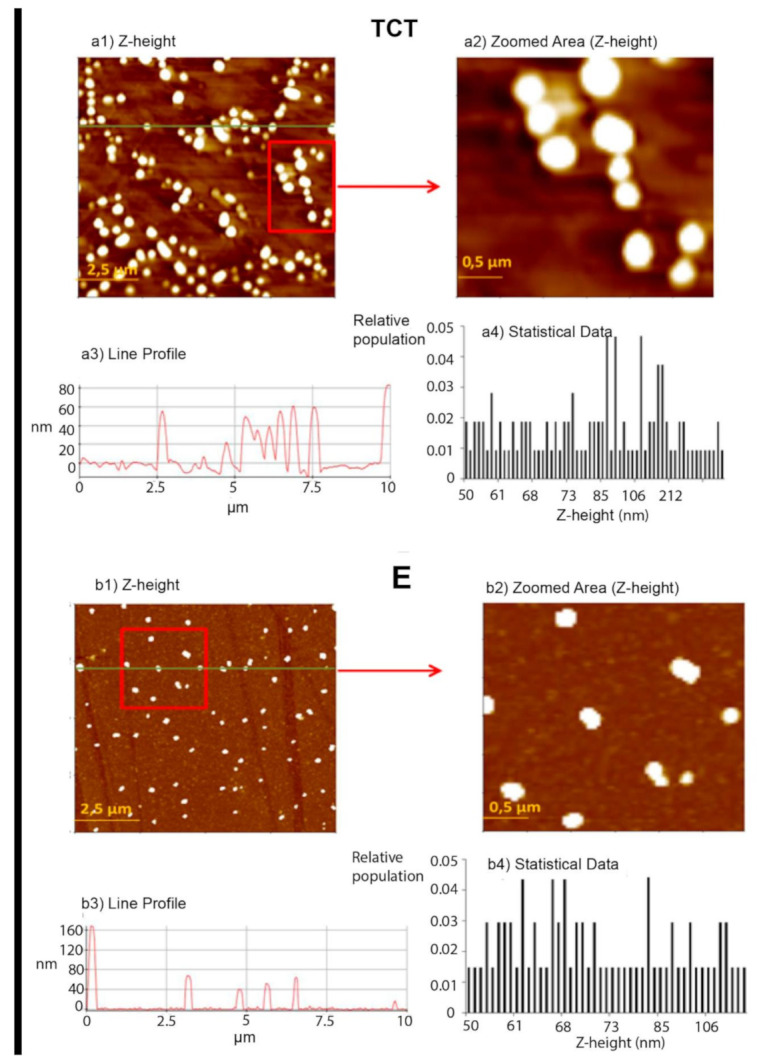
Representative images of EVs of TCT (**a**) and EVs of E (**b**) of *T. cruzi* Pan4 strain. (**a1**) Z-height image of EVs of TCT; (**a2**) zoomed area from image a1; (**a3**) line profile for the green line in the topography image (Z-height is in the range 40–80 nm) and (**a4**) statistical data of the relative frequency of the Z-height (nm). (**b1**) Z-height image of EVs of E; (**b2**) zoomed area from image b1; (**b3**) line profile for the green line in the topography image (Z-height is in the range 40–160 nm) and (**b4**) statistical data of the relative frequency of the Z-height (nm).

The morphology of EVs could be described as globular for both stages of the parasite. Although spanning a range generally between 1 and 10 nm (Figure 5(a1,b1)), the majority of these species have an average width of 20–90 nm in the case of TCT, and 30–113 nm in E. These results are in accordance with TEM and NTA results (Figure 1C–F).

AFM was employed to determine different nanomechanical parameters (Young modulus, stiffness and adhesion) of EVs of TCT and EVs of E of *T. cruzi* at the nanoscale level. Besides, AFM was also applied for assessing topographic measurements of EVs of both stages of the parasite. The morphology of EVs under TEM and AFM is shown in Figure 1 and Figure 5, respectively. Under our experimental conditions, significant amounts of EVs were obtained in both types of analysis. AFM analysis of freshly prepared EV samples revealed the presence of particles of heights between 50 and 200 nm for TCT and 50 and 160 nm for E, and correspond to monomeric species rather than aggregates (Figure 5a,b). Such observations further support the statistical data shown for both types of samples in the same figure.

##### Force Spectroscopy

**Figure 6:**
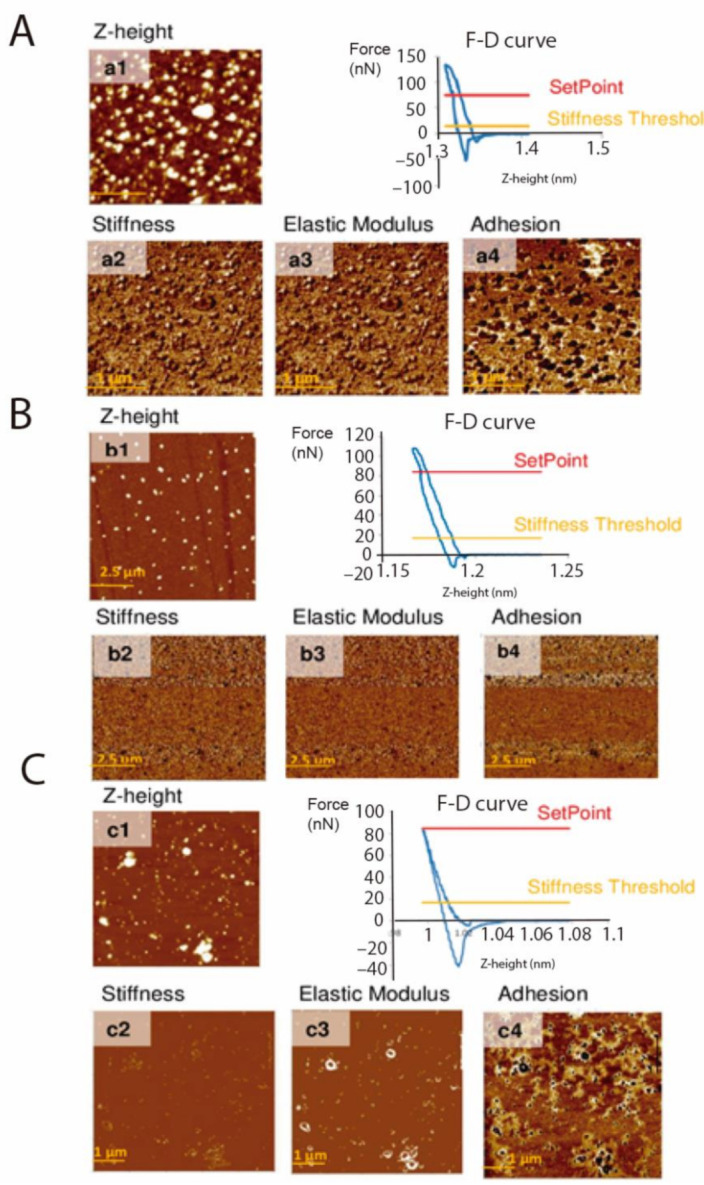
Atomic force microscopy Z-height images and nanomechanical maps showing stiffness, elastic modulus and adhesion profile of EVs of TCT (**A**), EVs of E (**B**) and EVs of TCT treated with proteinase K (**C**).

AFM was also employed for analyzing nanomechanical properties of EVs of E and TCT of *T. cruzi*, as previously mentioned. The purpose of including force spectroscopy studies was to compare nanomechanical properties for both types of samples isolated from different stages of the parasite: the elastic modulus, adhesion and stiffness. The measurements performed by AFM were carried out using the PinPoint mode, in which the tip touches the surface and is lifted after each pixel to improve friction and noise in the images, but result in F–D curves in different points of interest (vide infra Supplementary Materials. By using this mode, it is possible to study the details of material components and to perform distribution maps of material components. The analysis of the F–D curves allowed extracting the data regarding nanomechanical properties shown in this work (Figure 6).

Results revealed differences in the adhesion of EVs of E, compared to EVs of TCT (adhesion: 19.894 vs. 39.667 nN and 41.200 Nn when treated with proteinase K). On the other hand, assessment of stiffness and Young modulus showed a higher value in the case of EVs of E (20.471 N m^–1^ and 2.605 GPa, respectively) and EVs of TCT treated with proteinase K (57.581 N m^–1^ and 6.351 GPa, respectively), when compared to the values of EVs of TCT (13.913 N m^–1^ and 1.111 GPa, respectively) (Table 1). These results suggest that adhesion, as well as the stiffness and the elastic modulus of EVs are different between the two stages of the parasite and that the treatment of EVs of TCT with proteinase K modifies some of these properties.

#### 2.4.2. Measurement of zeta-potential (ZP)

To characterize the surface electrical charge of EVs of E and TCT of *T. cruzi,* ZP was measured in different samples. Determinations of the ZP of EVs treated with proteinase K (+PK), to remove proteins coating the vesicle; sodium periodate, to oxidize the carbohydrate of surface glycoproteins (+SP); and fetuin, a glycoprotein rich in sialic acid, were also included. Results are shown in Table 2.

## 3. Discussion

The quantity, surface molecular composition and dynamics of EVs released by cells changes upon the physiological status of the producer cell. Comparing the properties of the EVs released by two life cycle stages of a parasite, with different strategies for survival and thus, different communicators, is essential for understanding the adaptation of these unicellular organism to distinct environments within the insect vector or the mammalian host [29,30].

In 2010, Sharma et al. described EVs as structures with a non-homogeneous surface, tentatively attributable to the presence of either proteins, mRNA, or both, enclosed inside the lipid membrane [31]. Besides, Woo et al. (2016) confirmed the globular shapes of these extracellular vesicles [32] from mammalian cells, while comparing how different isolation methods affect the surface structure and size of EVs. To our knowledge, this is the first report regarding the characterization of EVs isolated from different stages of *T. cruzi—*including EVs submitted to different treatments/incubations—using AFM, a technology that exhibits substructural organization irresolvable by electron microscopy, including a description at the nanoscale level. Our results using AFM show the globular shape of the vesicles and confirm their integrity, in which intact, non-aggregated EVs were obtained after the isolation protocol described.

Additionally, a clear association in the protein composition of EVs of TCT and EVs of E (particularly of *trans*-sialidase proteins) and changes in adhesion, stiffness, elastic modulus, and zeta-potential was found. In this study, EVs of TCT showed higher adhesion values than EVs of E and the lowest stiffness and elastic modulus values. These results could suggest that proteins on the vesicle’s surface play an important role in these biophysical properties. In this sense, differential biophysical features of the EVs may be related to the glycocalyx of the EVs of TCT, which includes a dense layer of surface glycoproteins, many of them linked to GPI present in the membrane with highly adherent properties. Moreover, the vast majority of proteins found and enriched in EVs of TCT are predicted to be highly glycosylated and this fact would mediate interactions with lectins and receptors from the host cell membrane with carbohydrate residues, including sialic acid. Moreover, the lipid composition—mainly the amount of phospholipids and sterols—regulate the fluidity of the membrane. Therefore, differences found in the elastic modulus, together with the lower number of proteins present in the EVs of the epimastigote stage could be related to the higher content of sterol in the membrane of these forms [15]. Differential properties in stiffness and adhesion in metabolically different malignant (metastatic and non-metastatic) cell-derived EVs compared to non-malignant cells has been reported [33] and it is known that biomechanical properties of vesicles may play an important role in exocytosis and in intercellular transport [30].

The zeta-potential in a dispersed system is a measure of charge stability and affects particle-particle interactions [34]. As the electrical charge of the surface of EVs is reflected in the zeta-potential, it could be considered as a characteristic of the population of EVs. This electrical charge of the vesicles will depend, among others, on a series of factors: ionization of the components bound to the surface of the membrane, the chemistry of the grafted chains (if any), protonated states, inter and intramolecular bonds, presence of H bonds and the adsorption of ions of the electrolytes present in the solution in which they are found [33]. In our study, the slight differences shown in the standard deviation of the negative zeta-potential, higher in the case of EVs of E when compared to EVs of TCT, could indicate a wider distribution in the vesicles produced by these forms. Compatible with the size distribution observed by NTA, the samples of the EVs of E showed greater variability in both zeta-potential and size.

The sialic acid, once transferred from proteins sialylated by TS to the surface glycoproteins of the EVs, would increase the final electronegativity of EVs during the interaction with the cell membrane. As reported elsewhere, the zeta-potential of EVs of cancer cells was found to be higher than that of normal cells, and the large negative charge of cancer EVs was due to the large amount of sialic acids [35,36]. In a similar way to the slight differences found in EVs treated and untreated with proteinase K, the zeta-potential in EVs from cancer cells also show negative shifts compared to EVs from normal cells owing to abnormal expression of glycoprotein sugar chains due to malignant transformation which would mediate an increase in the adhesion properties of the vesicles [35]. Thus, the change observed after the reduction of sugars with sodium periodate would indicate that both proteins and oligosaccharides that are part of the glycocalyx of EVs with N-acetyl glucosamine O-linked oligosaccharides (that can be either sialylated by the parasite *trans*-sialidases or modified by α-galactose) could determine the net charge of these vesicles. Therefore, the differences in the electrostatic properties of EVs of *T. cruzi* could have outstanding importance since they may dictate the fate of the EVs of the parasite by targeting them to the plasma membrane of cells in tissues. Sialic-acid-rich regions would increase the negative charge on EVs as previously reported, if comparing the zeta-potential of EVs derived from cancer cells with or without the treatment with neuraminidase [35] and the increase in the electronegativity of EVs of TCT after being incubated with a glycosylated protein like fetuin, with three N-linked and three O-linked oligosaccharide chains, whose terminal sugar residues are rich in sialic acid [37]. The latter would imply the transfer of sialic acid from glycosylated proteins to sugars to the glycocalyx of the vesicles.

The proteome analysis of EVs of TCT and EVs of E indicate that the surface remodeling of the EVs described above is accompanied by a remarkable change in their protein cargos, particularly of TS surface proteins. *Trans*-sialidases are a highly polymorphic surface protein family (of around 1400–1700 genes, depending on the *T. cruzi* strain), divided into VIII groups, with only 15 genes encoding enzymes with potential catalytic activity [18,27]. The treatment of EVs with proteinase K clearly shows that TSs released via the exosomal pathway have a surface location. This suggests that the TS family of proteins will be in direct contact with the external milieu during the lifetime of the released EVs. It is noteworthy, that the percentage of TS proteins (22% vs. 7% in EVs of TCT and E, respectively) and the diversity of TS members (groups I–VI vs. group II in EVs of TCT and E, respectively) is remarkably increased in EVs of TCT. Due to the size (80–200 KDa) [38,39] and that they are heavily glycosylated, it could be suggested that they should have an impact on the different mechanical properties found in EVs of both stages.

Noteworthy, only the EVs from TCT carry TS proteins with catalytic activity (group I). Indeed, the proteomic analyses of the EVs of TCT have revealed the presence of the highly antigenic shed acute antigen (SAPA) belonging to TS group I, which presents C-terminal extensions which were suggested to protect the catalytic enzymatic activity as well as high reactivity of IgG and IgM antibodies against this protein [40]. In accordance with this data, previous works showed that E forms have a depletion of sialylated mucin acceptors [41], and a reduction transfer of sialylated products, since TS catalytic activity is 83% lower compared to the T stage [42]. The results found in the EVs of TCT would confirm the presence of TS proteins with a potential catalytic activity which would favor the propagation and survival of the T forms infecting forms via the removal of sialic acid from host cell donors that could be either exploited either via cell surface, EV release, or in combination. Physiological effects associated with this TS activity such as thrombocytopenia, apoptosis of immune cells in the spleen, thymus and peripheral lymph nodes [43,44] could be related to the action of enzymatically active TS on the surface of EVs of *T. cruzi*.

Group II of TS encompasses proteins related to adhesion and infection in the trypomastigote stage, such as SA-1, SA85, gp90, gp82 and ASP-2 [27]. Another group of TS proteins described in the proteome of the EVs of TCT with known activity are the complement regulatory proteins (CRP) (TS group III) that inhibits alternative and classical pathways of complement activation [45,46], thus providing these EVs with a potential role in evading immune system targets in the parasite [6,25,47]. Moreover, EVs of TCT also displayed Tc13 (TS group IV), which is highly expressed in metacyclic and TCT and are able to bind β2-adrenergic receptors and modify myocardial contractile activity [6,48,49,50]. In this sense, the EVs of TCT have shown the ability to interact with this receptor [8], a fact that may be mediated by the exposure of the Tc13 protein on the surface of EVs. The functionality of all other members of the proteome of the EVs of T forms falling in the other TS groups remains poorly characterized; however, one might think that the polymorphic structure of TS family will confer a high diversity of roles for the EVs secreted by trypomastigotes.

Other difference arose after analyzing TS protein expression and chromosome mapping, in which TS exposed over the surface of the EVs of TCT have a random distribution across the genome, with the exception of group II of TS. In this regard, specific associations of class II TS of EVs of E (and TS class II shared with EVs of TCT) with telomeric regions have been found and this could indicate a precise regulation of (I) protein expression or (II) protein location and cargos through the EVs’ compartments. As commented on above, group II of TS has been extensively studied and described and they have functions related to host-parasite interactions thus exposed to high environmental pressures, which may require constant rearrangements at the subtelomeric regions as suggested by Freitas et al. [27]. Interestingly, the finding of this regulatory pathway could be explained by differential regulation due to differences in TS UTRs between TCT and E [27]. Here, the expression of all TS groups in the EVs of TCT is correlated to the data reported by Freitas et al. [27], where they show that all TS groups have potential antigenic properties when exposed to sera from infected mice and thus expressed at some point by the trypomastigote stage.

## 4. Materials and Methods

### 4.1. Cell Culture, Parasite Strain and Stages

Vero cells (ECACC 84113001) were cultured in Nunc cell-culture flasks of 75 cm^2^ surface area (Thermo Fischer Scientific, Waltham, MA, USA) in Minimum Essential Medium (MEM) (Sigma Aldrich, St. Louis, MO, USA) supplemented with 10% fetal bovine serum (Gibco, Waltham, MA, USA) previously inactivated at 56 °C for 30 min (iFBS), plus antibiotics (penicillin 100 U/mL, streptomycin 100 μg/mL). The cultures were maintained at 37 °C, in a moist atmosphere enriched with 5% CO_2_.

*Trypanosoma cruzi* Pan4 strain (TcIa + TcId) was employed in this work. This strain was isolated from a patient from Panama during 2004. The epimastigote stage of the parasite was cultured in liver infusion tryptose (LIT) medium supplemented with 10% heat-inactivated fetal bovine serum, at 28 °C. Trypomastigote forms derived from cell cultures were obtained from infected Vero cell cultures as previously described [51,52,53,54].

### 4.2. Isolation and Purification of EVs of TCT and EVs of E of T. cruzi

#### 4.2.1. Isolation of EVs

Trypomastigotes of the Pan4 strain were obtained after the infection of cultured Vero cells with purified metacyclic trypomastigotes. The infections were performed in Nunc cell culture flasks of 75 cm^2^ surface area with MEM supplemented with 10% IFBS plus penicillin (100 U/mL) and streptomycin (100 µg/ml), and maintained at 37 °C and 5% CO_2_. After 120 h of the intracellular development of the parasite, the culture media containing the trypomastigote forms derived from cell cultures were collected and centrifuged at 3000× *g* for 5 min. The pellet with trypomastigotes was washed in sterile phosphate buffer saline (PBS) four times.

The purification of EVs from TCT and E was performed following the methodology previously described by Díaz Lozano et al. [51] and Retana Moreira et al. [8]. Briefly, 5 × 10^7^ TCT of *T. cruzi* were incubated for 5 h in 5 ml Roswell Park Memorial Institute (RPMI) 1640 medium (Sigma Aldrich, St. Louis, MO, USA) buffered with 25 mM HEPES, pH 7.2, and supplemented with 10% exosome-free iFBS obtained after centrifugation at 100,000× *g* during 16–18 h. After this time, the parasites were removed by centrifugation at 3500× *g* for 15 min and the supernatant was collected and centrifuged at 17,000× *g* for 30 min at 4 °C, in order to remove eventual apoptotic blebs and cell debris. The 17,000× *g* supernatant was filtered through a 0.22 µm pore filter (Sartorius, Göttingen, Germany) and ultracentrifuged at 100,000× *g* for 16–18 h in an ultracentrifuge Avanti J-301 (Beckman Coulter, Brea, CA, USA) with a JA-30.50 Ti rotor. The resulting pellet was washed three times in sterile PBS in an ultracentrifuge Sorwal WX80 (Thermo Fisher Scientific, Waltham, MA, USA) with F50L-24 × 1.5 fixed-angle rotor and resuspended in 100 µl PBS. The viability of the TCT after shedding of EVs was evaluated using the trypan blue exclusion test. After 5 h, no significant death was detected and over 99% of the parasites were viable.

For the isolation of EVs from E, 5 × 10^7^ parasites were incubated for 5 h at 28 °C in LIT culture medium with 10% exosome-free IFBS. After a 5 h incubation, the parasites were centrifuged at 3500× *g* for 15 min and the supernatant was submitted to the protocol recently described for the isolation of EVs from TCT. The EVs were washed 3 times in PBS, resuspended in 100 µl PBS plus cOmplete, ethylenediamine tetraacetic acid (EDTA)-free protease inhibitor cocktail (Roche, Basel, Switzerland) and stored at −80 °C. The isolation procedure is summarized in Supplementary Figure S1. This figure contains images and graphs of the size of the vesicles from the pellets of the purification process.

The isolation procedure was evaluated by transmission electron microscopy, atomic force microscopy, dynamic light scattering and nanoparticle tracking analysis. The protein concentration from each sample of EVs was quantified using the Micro-BCA protein assay kit (Thermo Fischer Scientific, Waltham, MA, USA), as described elsewhere [8]. For the proteomic analysis, the incubations of each stage of the parasite were performed in culture media without iFBS and the protein concentration obtained under the conditions described above were 13.1 µg for EVs of E and 16.7 µg for EVs of TCT.

#### 4.2.2. Transmission Electron Microscopy

For TEM, EVs obtained as described above were fixed in Karnovsky’s fixative [30] (2.5% glutaraldehyde (*v/v*) and 2% formaldehyde (*v/v*) in 0.1 M cacodylate buffer; 50 mg of CaCl_2_ in 100 ml) at 37 °C for 30 min, dehydrated and embedded in Spurr resin (Sigma, Ronkonkoma, NY, USA). After performing the ultra-thin sections, they were stained with 8% uranyl acetate (vol/vol) followed by 0.2% lead citrate (*v/v*) and visualized on a Carl Zeiss LIBRA 120 PLUS SMT electron microscope.

Samples of EVs treated with proteinase K were negative stained and submitted to immunogold labeling for the detection of *trans*-sialidases using the anti-*trans*-sialidase antibody (mAb 39). In this case, aliquots of the purified EVs were resuspended in 30 µl Tris-HCl (pH 7.3) and 5 µl of the suspension was adsorbed directly onto Formvar/carbon-coated grids. After 30 min, the grids were washed three times for 10 min in PBS and fixed in 1% glutaraldehyde for 30 min. The grids were then washed three times in PBS, blocked for 30 min in a solution of 0.02 M glycine prepared in 0.1 M PBS (pH 7.3) and blocked for 30 min with blocking buffer (0.05% Tween in PBS, pH 7.4, plus 1% nonfat milk). After this time, the grids were incubated with the anti-*trans*-sialidase antibody (mAb 39) (1:50) and the anti-mouse IgG (whole molecule)-gold (20 nm) labeled antibody (Sigma-Aldrich, St. Louis, MO, USA) for 2 h for each incubation. The final contrast was performed with 2% (*v/v*) uranyl acetate, as previously reported [53,54].

#### 4.2.3. Dynamic Light Scattering and Nanoparticle Tracking Analysis

DLS analysis of isolated EVs of TCT and EVs of E were performed using a Zetasizer nano ZN90 (Malvern Panalytical, Worcestershire, UK) and measured at 25 °C. For data acquisition and information processing, Zetasizer Ver. 7.11 software was employed.

The hydrodynamic size distribution of the purified EVs was measured by NTA, using an instrument equipped with a sample chamber, a 405-nm laser and a high-sensitivity complementary metal-oxide-semiconductor (CMOS) camera. The samples were diluted in 0.22 μm filtered PBS up to 1 ml and then, loaded into the chamber. Three 60 s videos, in Brownian mode, were recorded and analyzed for each sample with NTA 2.3 image-analysis software (NanoSight Ltd., Amesbury, UK). Measurement conditions were manual shutter, gain, brightness and threshold adjustments at 25 °C. The mean size distribution was calculated as a mean of three independent size distributions.

### 4.3. Scanning Electron Microscopy of TCT and E

SEM was performed according to Díaz Lozano et al. [51]. Briefly, the parasites were placed over 13-mm round coverslips (Marienfeld, Lauda-Königshofen, Germany), in Nunc 24-well plates (Thermo Fischer Scientific, Waltham, MA, USA). After 4 h, the coverslips were fixed with 2.5% glutaraldehyde in cacodylate buffer with 0.1 M saccharose and maintained in the fixative solution for 24 h at 4 °C. After this time, the samples were dehydrated in a graded series of ethanol, desiccated using a critical point dryer (LEICA EM CPD 300) and evaporated with a high vacuum carbon coater (EMITECH K975X). Finally, the samples were carbon-coated for 3 min and observed in a ZEISS Supra microscope.

### 4.4. Enzymatical and Chemical Treatment of EVs

EVs (40 μg) of TCT of *T. cruzi* were treated with proteinase K (final concentration 0.5 mg/ml) (Sigma-Aldrich, St. Louis, MO, USA) for 30 min at 37 °C in agitation, according to Retana Moreira et al. (2019). The suspension was then washed twice performing 100,000× *g* ultracentrifugation steps for 4 h, removing the supernatant containing the proteinase K. The pellet with EVs was then resuspended in 0.22 μm filtered PBS with protease inhibitors (cOmplete, EDTA-free protease inhibitor cocktail) (Roche, Basel, Switzerland).

To reduce the glycoconjugates surrounding the EVs, treatment with sodium periodate was applied. In this case, EVs were incubated with 10 mg/ml of the reagent for 90 min at room temperature in the dark. After the incubation, EVs were washed as described above. Samples of EVs of TCT were also incubated in a solution containing 100 µg/ml fetuin, a glycosylated protein rich in sialic acid (N-acetylneuraminic acid), in MEM, for 90 min. The samples were subsequently washed twice.

### 4.5. Proteomic Analyses of EVs from TCT and E

Samples of EVs were concentrated by adding 4 volumes of cold acetone to each sample and incubating overnight at −20 °C. After this time, acetone was removed by centrifugation at 13,000× *g* for 10 min at 4 °C and two washing steps in 1 ml of cold acetone were performed. The acetone was removed with a mixture of nitrogen and air stream and the samples were stored dried at −80 °C until proteomic analyses.

Protein extracts from EVs of 4 biological replicates of TCT and 3 biological replicates of E (40 µg/sample) were resuspended in a volume up to 50 µl of sample buffer, and then applied onto 1.2-cm wide wells of a conventional SDS-PAGE gel (0.75 mm thick, 4% stacking and 10% resolving). The run was stopped as soon as the front entered 3 mm into the resolving gel, so that the whole proteome became concentrated in the stacking/resolving gel interface. The unseparated protein bands were visualized by Coomassie staining, excised, cut into cubes (2 × 2 mm), and placed in 0.5 ml microcentrifuge tubes [55]. The gel pieces were destained in acetonitrile:water (ACN:H_2_O, 1:1) and disulfide bonds from cysteinyl residues were reduced with 10 mM dithiothreitol (DTT) for 1 h at 56 °C. Then, thiol groups were alkylated with 50 mM iodoacetamide for 1 h at room temperature in the darkness and digested in situ with sequencing grade trypsin (Promega, Madison, WI, USA) as described by Shevchenko et al. in 1996 with minor modifications [56]. The gel pieces were shrunk by removing all liquid using sufficient ACN. Acetonitrile was pipetted out and the gel pieces were dried in a SpeedVac™. The dried gel pieces were re-swollen in 50 mM ammonium bicarbonate pH 8.8 with 60 ng/µl trypsin at 5:1 protein:trypsin (*w/w*) ratio. The tubes were kept in ice for 2 h and incubated at 37 °C for 12 h. Digestion was stopped by the addition of 1% trifluoroacetic acid (TFA). Whole supernatants were dried down and then desalted onto ZipTip C18 pipette tips (Millipore), OMIX pipette tips C18 (Agilent Technologies, Santa Clara, CA, USA) or OASIS C18 columns (Waters) until mass spectrometric analysis.

The desalted protein digest was dried, resuspended in 10 µL of 0.1% formic acid and analyzed by reversed-phase liquid chromatography with tandem mass spectrometry (RP-LC-MS/MS) in an Easy-nLC II system coupled to an ion trap LTQOrbitrap-Velos-Pro hybrid mass spectrometer (Thermo Fischer Scientific, Waltham, MA, USA). The peptides were concentrated (on-line) by reverse phase chromatography using a 0.1 mm × 20 mm C18 RP precolumn (Thermo Fischer Scientific, Waltham, MA, USA), and then separated using a 0.075 mm × 250 mm C18 RP column (Thermo Fischer Scientific, Waltham, MA, USA) operating at 0.3 μl/min. Peptides were eluted using a 240-min dual gradient from 5 to 25% solvent B for 180 min followed by a gradient from 25 to 40% solvent B over 240 min (Solvent A: 0.1% formic acid in water; solvent B: 0.1% formic acid, 80% acetonitrile in water). Electrospray ionization (ESI) ionization was performed using a Nanobore emitters Stainless Steel ID 30 μm (Proxeon) interface. The Orbitrap resolution was set at 30,000. Peptides were detected in survey scans from 400 to 1600 amu (1 μscan), followed by fifteen data-dependent MS/MS scans (Top 15), using an isolation width of 2 u (in mass to-charge ratio units), normalized collision energy of 35%, and dynamic exclusion applied during 30 s periods.

Peptide identification from raw data was carried out using the SEQUEST algorithm (Proteome Discoverer 1.4, Thermo Fischer Scientific, Waltham, MA, USA). A database search was performed (UniProt-*Trypanosoma cruzi*_CL Brener, Uniprot-*Bos taurus* y Uniprot-*Chlorocebus sabaeus*). The following constraints were used for the searches: tryptic cleavage after Arg and Lys, up to two missed cleavage sites and tolerances of 10 ppm for precursor ions and 0.8 Da for MS/MS fragment ions, and the searches were performed allowing optional Met oxidation and Cys carbamidomethylation. A search was performed against the decoy database (integrated decoy approach) using a false discovery rate (FDR) < 0.01 [57]

### 4.6. Bioinformatic Analyses

Protein enrichments were classified according to Gene Ontology (GO) with the trytrip GO terms enrichment tool and searching for molecular function ontology using *T. cruzi* CL Brener strain as reference organism and Bonferroni correction with a cut-off value of *p* ≥ 0.01 and represented as scatter plots and interactive graphs using REVIGO web tool (http://revigo.irb.hr/, accessed on 27 June 2017). The terms GO:0003674 “molecular function” and GO:0005198 “structural molecule activity” terms were not represented.

To generate a chromosomal distribution of EVs’ TS groups we followed the methodology described by Freitas et al. (2011) [27]. Briefly, the chromosomal coordinates of the TS genes were retrieved using *T. cruzi* CL-Brener genome from TriTrypDB (http://TriTrypDB.org, accessed on 27 June 2017) and plotted on the chromosomes.

For each protein, the probability of appearing in a given proteome sample type was estimated by dividing the number of samples in which the protein was found by the total number of samples of that proteome sample type. To show the level of precision in the estimate, we accompanied these values with confidence intervals. In particular, we calculated the modified Wilson confidence interval for a binomial proportion using the BinomCI function from the R DescTools package. Violin plots were generated using the ggplot2 package.

### 4.7. Production of Polyclonal Anti-T. cruzi Antibodies

Five male BALB/c mice were immunized with 20 µg of a total extract of purified trypomastigotes of the *T. cruzi* Pan4 strain to produce an anti-*T. cruzi* antibody. A suspension of the antigen was prepared by dilution of the lysed parasites in PSB plus Freund’s adjuvant (Sigma, Ronkonkoma, NY, USA), in a 1:1 ratio (final volume: 500 µl). This suspension was administered intraperitoneally to mice and, for subsequent immunizations, the adjuvant was then switched to incomplete Freund’s adjuvant (Sigma-Aldrich, St. Louis, MO, USA). A total of 7 immunizations (1 per week) were performed and the animals were exsanguinated two weeks after the final immunization. The antibody production was evaluated using ELISA and Western blot (WB), as previously described [8,58].

### 4.8. Western Blotting

To confirm the presence of specific proteins in EVs of TCT and EVs of E identified in the proteomic analysis, Western blots using anti-cruzipain and anti-*trans*-sialidase (mAb39) antibodies (as described by Retana Moreira et al. in 2019) [8] and the anti-*T. cruzi* antibody obtained after the immunization of mice (described in the previous section) were performed. For each WB, 30 µg of EVs of TCT or E were resolved by SDS-PAGE, transferred to a nitrocellulose membrane and blocked overnight with 5% non-fat milk in PBS-0.1% Tween 20. The membranes were then washed in PBS-0.1% Tween 20 and incubated overnight at 4 °C with different primary antibodies: anti-cruzipain (1:1000) (produced in rabbit), anti-TS mAb 39 (1:1000) (produced in mice) and anti-*T. cruzi* (1:100) (produced in mice). After incubation, the membranes were washed again in PBS-0.1% Tween 20 and incubated for 1 h with goat anti-rabbit IgGs conjugated with peroxidase (1:2000) in the case of cruzipain (Agilent Technologies, Satna Clara, CA, USA) and goat anti-mouse IgGs conjugated with peroxidase (1:1000) (Agilent Technologies, Santa Clara, CA, USA) in the case of TS and anti-*T. cruzi.* The reaction was visualized using Clarity ECL Western substrate (BioRad, Hercules, CA, USA) in a ChemiDoc Imaging System (BioRad, Hercules, CA, USA).

### 4.9. Measurement of Zeta-potential of EVs

To determine the zeta-potential of EVs of *T. cruzi*, samples of EVs of TCT and EVs of E, as well as samples of EVs treated as described in Section 4.4, were resuspended in phoshate buffer (PB) 7.4, 1.13 mM, 10 mM NaCl solution and the electrophoretic mobility (μe) was measured by Laser Doppler electrophoresis, in a Zetasizer NanoZeta ZS (Malvern Panalytical, Worcestershire, UK), at 25 °C. ZP was calculated by using the Smoluchowski equation and results were obtained from at least three independent measures.

### 4.10. Atomic Force Microscopy

#### 4.10.1. Topographic Measurements and Imaging

To investigate the morphology of EVs of TCT and EVs of E, non-contact mode AFM imaging was performed using an NX-20 instrument (Park Systems, Suwon, Korea) and ACTA cantilevers (K = 40 N m^–1^ and *f* = 320 kHz). Each sample was diluted 1:4 in sterile-filtered PBS to obtain a concentration of 1.55 µg/µL. A volume of 8 µL of the dilution of EVs was deposited onto freshly cleaved muscovite mica, the most common substrate for single-molecule imaging of DNA and proteins and also considered the first choice for AFM imaging of extracellular vesicles [59]. The samples were deposited on the substrate for 10–15 min and they were rinsed three times with MilliQ water (Millipore, Burlington, MA, USA) to remove salts and loosely bound EVs. Finally, the samples over the mica substrates were further dried before imaging with a gentle stream of argon. Images were typically acquired as 256 × 256 pixels at a scan rate of 0.5–0.7 Hz. Subsequently, images were processed and analyzed using XEI software (Park Systems, Suwon, Korea). Representative images of samples were obtained by scanning at least 3 different locations on at least 3 different samples of the same nature.

#### 4.10.2. Force Spectroscopy

For the determination of the nanomechanical properties of EVs of each stage of the parasite, the samples were diluted 1:100 in PBS and deposited onto freshly cleaved muscovite mica sheets for 10 min. After the adsorption process, mica sheets were then rinsed with deionized water and dried under a gentle stream of argon, as described previously. In this case, NSC-14 probes were used (K = 5 N m^–1^ and *f* = 160 kHz) and measurements were performed using the gentle PinPoint mode to acquire reproducible and reliable topography, stiffness, adhesion and elastic module maps. The measurement procedure could be explained in 3 steps: (1) the XY scanner stops during acquisition; (2) the tip approaches the surface, measures mechanical properties and retracts from the surface over a few ms (4 ms) to achieve an interaction force preset (15 nN) and (3) records the approach height maintaining the Z distance. With this procedure, the force is held constant while properties are measured, then the tip is retracted and moved to the next pixel (Supplementary Materials and Figure S6).

## 5. Conclusions

This work clearly shows that the developmental differentiation from E to eventual TCT will impact properties of EVs at different levels: protein composition, biophysical parameters (adhesion, Young modulus and stiffness) and the surface electric charge. The differences observed suggest that EVs of TCT are more prone to withstand changes in length when under lengthwise tension or compression with a polymorphic glycoprotein surface, a reflection of the high variety of environments faced by trypomastigotes in the mammalian hosts, increasing its chances for survival.

## Figures and Tables

**Table 1:** Nanomechanical properties of EVs of TCT, EVs of E and EVs of TCT after the treatment with proteinase K *.

Sample Type	Stiffness (N m^–1^)	Elastic Modulus (GPa)	Adhesion (nN)
EVs of TCT	13.913 ± 02.783	1.111 ± 0.218	39.667 ± 07.140
EVs of E	20.471 ± 04.709	2.605 ± 0.706	19.894 ± 02.163
EVs of TCT +PK	57.581 ± 05.043	6.351 ± 0.896	41.200 ± 04.293

* For each type of sample, measurements were repeated in 3 different zones and F–D curves were recorded for each pixel, of which 10 different points per zone have been assessed. This assumes a total of 30 different values averaged to give the final results indicated in the table.

**Table 2:** Zeta-potential of EVs of TCT, EVs of E and EVs of TCT after the treatment/incubation with different reagents/proteins *.

Sample Type	Zeta-Potential (mV)
EVs of TCT	–16 ± 4
EVs of E	–18 ± 8
EVs of TCT + PK	–18 ± 6
EVs of TCT + SP	–17 ± 4
EVs of TCT + fetuin	–19 ± 5

* Results consist of the average of 3 ZP values, obtained after 3 different measurements by the instrument. The corresponding error is the mean of the errors of each of these independent measurements, which is obtained as a standard deviation of the zeta potential distribution resulting from that measurement. In turn, each ZP measurement given by the instrument is the mean of a ZP distribution (the corresponding error is the standard deviation) obtained by the software from 3 internal measurements and each of these internal measurements is at its own expense.

## Data Availability

Mass spectrometry data along with the identification results have been deposited to the ProteomeXchange Consortium via the PRIDE partner repository (DOI:10.1093/nar/gks1262) with the dataset identifiers PXD013875, PXD013876, PXD013878, PXD013880, PXD013882, PXD013901 and 10.6019/PXD013875, 10.6019/PXD013876, 10.6019/PXD013878, 10.6019/PXD013880, 10.6019/PXD013882, 10.6019/PXD013901.

## Supplementary Materials

The following are available online at https://www.mdpi.com/article/10.3390/ijms22105183/s1, Figure S1: Schematic representation of the isolation and purification of EVs of *T. cruzi* Pan4 strain, Table S1: Proteins identified in EVs of TCT and EVs of E, Figure S2: Protein richness and frequency in EVs of *T. cruzi,* Table S2: Differential enrichment in GO terms in EVs of TCT and EVs of E, Figure S3: Venn diagram for the comparison of the cargo of proteins of EVs of E and EVs of TCT of *T. cruzi* Pan4 strain employed in this study and the cargo of proteins of the EVs of E and MT stages isolated by Bayer Santos et al. in 2013, Figure S4: Percentage of appearance of TS in EVs of *T. cruzi*: TS protein richness and appearance frequency, Figure S5: F–D curve and parameters for nanomechanical properties.

## References

[1] Van der Pol E., Böing A.N., Harrison P., Sturk A., Nieuwland R. (2012). Classification, functions, and clinical relevance of extracellular vesicles. Pharmacol. Rev..

[2] Bayer Santos E., Lima F.M., Ruiz J.C., Almeida I.C., da Silveira J.F. (2014). Characterization of the small RNA content of *Trypanosoma cruzi* extracellular vesicles. Mol. Biochem. Parasitol..

[3] Raposo G., Stoorvogel W. (2013). Extracellular vesicles: Exosomes, microvesicles, and friends. J. Cell Biol..

[4] Wang J., Sun X., Zhao J., Yang Y., Cai X., Xu J., Cao P. (2017). Exosomes: A novel strategy for treatment and prevention of diseases. Front. Pharmacol..

[5] Marcilla A., Martin Jaular L., Trelis M., de Menezes Neto A., Osuna A., Bernal D., Fernandez Becerra C., Almeida, Igor C., del Portillo H.A. (2014). Extracellular vesicles in parasitic diseases. J. Extracell. Vesicles..

[6] De Pablos L.M., Retana Moreira L., Osuna A. (2018). Exovesicles in Chagas disease: New passengers for an old disease. Front. Microbiol..

[7] Ofir Birin Y., Heidenreich M., Regev Rudzki N. (2017). Pathogen-derived extracellular vesicles coordinate social behaviour and host manipulation. Semin. Cell Dev. Biol..

[8] Retana Moreira L., Rodríguez Serrano F., Osuna A. (2019). Extracellular vesicles of *Trypanosoma cruzi*: Induction of physiological changes in non-parasitized culture cells. PLoS Negl. Trop. Dis..

[9] Silverman J.M., Clos J., de’Oliveira C.C., Shirvani O., Fang Y., Wang C., Foster L.J., Reiner N.E. (2010). An exosome-based secretion pathway is responsible for protein export from *Leishmania* and communication with macrophages. J. Cell Sci..

[10] Atayde V.D., Aslan H., Townsend S., Hassani K., Kamhawi S., Olivier M. (2015). Exosome secretion by the parasitic protozoan *Leishmania* within the sand fly midgut. Cell Rep..

[11] World Health Organization (WHO) (2017). Chagas Disease. http://www.who.int/mediacentre/factsheets/fs340/en/.

[12] Da Silveira J., Abrahamsohn P., Colli W. (1979). Plasma membrane vesicles isolated from epimastigote forms of *Trypanosoma cruzi*. Biochim. Biophys. Acta.

[13] Gonçalves R.L., Barreto R.F., Polycarpo C.R., Gadelha F.R., Castro S.L., Oliveira M.F. (2011). A comparative assessment of mitochondrial function in epimastigotes and bloodstream trypomastigotes of *Trypanosoma cruzi*. J. Bioenerg. Biomembr..

[14] Kaneda Y., Nagakura K., Goutsu T. (1986). Lipid composition of three morphological stages of *Trypanosoma cruzi*. Comp. Biochem. Physiol..

[15] Sharma A.I., Olson C.L., Mamede J.I., Gazos-Lopes F., Epting C.L., Almeida I.C., Engman D.M. (2017). Sterol targeting drugs reveal life cycle stage-specific differences in trypanosome lipid rafts. Sci. Rep..

[16] Li Y., Shah-Simpson S., Okrah K., Belew A.T., Choi J., Caradonna K.L., Padmanabhan P., Ndegwa D.M., Temanni M.R., Corrada Bravo H. (2016). Transcriptome remodeling in *Trypanosoma cruzi* and human cells during intracellular infection. PLoS Pathog..

[17] Alves M.J., Kawahara R., Viner R., Colli W., Mattos E.C., Thaysen-Andersen M., Larsen M.R., Palmisano G. (2017). Comprehensive glycoprofiling of the epimastigote and trypomastigote stages of *Trypanosoma cruzi*. J. Proteom..

[18] Atwood J.A. (2005). The *Trypanosoma cruzi* proteome. Science.

[19] Schenkman S., Ferguson M.A., Heise N., de Almeida M.L., Mortara R.A., Yoshida N. (1993). Mucin-like glycoproteins linked to the membrane by glycosylphosphatidylinositol anchor are the major acceptors of sialic acid in a reaction catalyzed by trans-sialidase in metacyclic forms of *Trypanosoma cruzi*. Mol. Biochem. Parasitol..

[20] San Francisco J., Barría I., Gutiérrez B., Neira I., Muñoz C., Sagua H., Araya J.E., Andrade J.C., Zailberger A., Catalán A. (2017). Decreased cruzipain and gp85/trans-sialidase family protein expression contributes to loss of *Trypanosoma cruzi* trypomastigote virulence. Microbes Infect..

[21] Tonelli R.R., Torrecilhas A.C., Jacysyn J.F., Juliano M.A., Colli W., Alves M.J. (2011). In vivo infection by *Trypanosoma cruzi*: The conserved FLY domain of the gp85/trans-sialidase family potentiates host infection. Parasitology.

[22] Freire de Lima L., Fonseca L., Oeltmann T., Mendonça Previato L., Previato J. (2015). The *trans*-sialidase, the major *Trypanosoma cruzi* virulence factor: Three decades of studies. Glycobiology.

[23] Lantos A.B., Carlevaro G., Araoz B., Ruiz Diaz P., Camara Mde L., Buscaglia C.A., Bossi M., Yu H., Chen X., Bertozzi C.R. (2016). Sialic acid glycobiology unveils *Trypanosoma cruzi* trypomastigote membrane physiology. PLoS Pathog..

[24] Bayer Santos E. (2013). Proteomic analysis of *Trypanosoma cruzi* secretome: Characterization of two populations of extracellular vesicles and soluble proteins. J. Proteome Res..

[25] Cestari I., Ansa Addo E., Deolindo P., Inal J.M., Ramirez M.I. (2012). *Trypanosoma cruzi* immune evasion mediated by host cell-derived microvesicles. J. Immunol..

[26] Ramirez M.I., Deolindo P., de Messias-Reason I.J., Arigi E.A., Choi H., Almeida I.C., Evans-Osses I. (2016). Dynamic flux of microvesicles modulate parasite-host cell interaction of *Trypanosoma cruzi* in eukaryotic cells. Cell. Microbiol..

[27] Freitas L.M., dos Santos S.L., Rodrigues-Luiz G.F., Mendes T.A., Rodrigues T.S., Gazzinelli R.T., Teixeira S.M., Fujiwara R.T., Bartholomeu D.C. (2011). Genomic analyses, gene expression and antigenic profile of the trans-sialidase superfamily of *Trypanosoma cruzi* reveal an undetected level of complexity. PLoS ONE.

[28] Schenkman S., Pontes de Carvalho L., Nussenzweig V. (1992). Trypanosoma cruzi trans-sialidase and neuraminidase activities can be mediated by the same enzymes. J. Exp. Med..

[29] Akers J.C., Gonda D., Kim R., Carter B.S., Chen C.C. (2013). Biogenesis of extracellular vesicles (EV): Exosomes, microvesicles, retrovirus-like vesicles, and apoptotic bodies. J. Neuro Oncol..

[30] Théry C., Ostrowski M., Segura E. (2009). Membrane vesicles as conveyors of immune responses. Nat. Rev. Immunol..

[31] Sharma S., Rasool H.I., Palanisamy V., Mathisen C., Schmidt M., Wong D.T., Gimzewski J.K. (2010). Structural-mechanical characterization of nanoparticle exosomes in human saliva, using correlative AFM, FESEM, and Force Spectroscopy. ACS Nano.

[32] Woo J., Sharma S., Gimzewski J. (2016). The role of isolation methods on a nanoscale surface structure and its effect on the size of exosomes. J. Circ. Biomark..

[33] Midekessa G., Godakumara K., Ord J., Viil J., Lättekivi F., Dissanayake K., Kopanchuk S., Rinken A., Andronowska A., Bhattacharjee S. (2020). Zeta potential of extracellular vesicles: Toward understanding the attributes that determine colloidal stability. ACS Omega.

[34] Beit Yannai E., Tabak S., Stamer W.D. (2018). Physical exosome:exosome interactions. J. Cell. Mol. Med..

[35] Akagi T., Ichiki T., Ohshima H. (2016). Evaluation of Zeta potential of individual exosomes secreted from biological cells using a microcapillary electrophoresis Chip. Encycl. Biocolloid Biointerface Sci..

[36] Whitehead B., Wu L., Hvam M.L., Aslan H., Dong M., Dyrskjøt L., Ostenfeld M.S., Moghimi S.M., Howard K.A. (2015). Tumour exosomes display differential mechanical and complement activation properties dependent on malignant state: Implications in endothelial leakiness. J. Extracell. Vesicles.

[37] Wang H., Zhang M., Bianchi M., Sherry B., Sama A., Tracey K.J. (1998). Fetuin (alpha2-HS-glycoprotein) opsonizes cationic macrophage deactivating molecules. Proc. Natl. Acad. Sci. USA.

[38] Torrecilhas A.C., Soares R.P., Schenkman S., Fernández Prada C., Olivier M. (2020). Extracellular vesicles in trypanosomatids: Host cell communication. Front. Cell. Infect. Microbiol..

[39] El-Sayed N.M., Myler P.J., Bartholomeu D.C., Nilsson D., Aggarwal G., Tran A.N., Ghedin E., Worthey E.A., Delcher A.L., Blandin G. (2005). The genome sequence of *Trypanosoma cruzi*, etiologic agent of Chagas disease. Science.

[40] Herreros Cabello A., Callejas Hernández F., Gironès N., Fresno M. (2020). Trypanosoma Cruzi genome: Organization, multi-gene families, transcription, and biological implications. Genes.

[41] Schenkman S., Eichinger D., Pereira M.E., Nussenzweig V. (1994). Structural and functional properties of *Trypanosoma* trans-sialidase. Annu. Rev. Microbiol..

[42] Urban I., Santurio L.B., Chidichimo A., Yu H., Chen X., Mucci J., Agüero F., Buscaglia C.A. (2011). Molecular diversity of the *Trypanosoma cruzi* TcSMUG family of mucin genes and proteins. Biochem. J..

[43] De Lederkremer R.M., Agusti R. (2009). Chapter 7 Glycobiology of *Trypanosoma cruzi*. Adv. Carbohydr. Chem. Biochem..

[44] Tribulatti M.V., Mucci J., Van Rooijen N., Leguizamon M.S., Campetella O. (2005). The *Trans*-sialidase from *Trypanosoma cruzi* induces thrombocytopenia during acute Chagas’ disease by reducing the platelet sialic acid contents. Infect. Immun..

[45] De Pablos L.M., Osuna A. (2012). multigene families in *Trypanosoma cruzi* and their role in infectivity. Infect. Immun..

[46] Beucher M., Norris K.A. (2008). Sequence diversity of the *Trypanosoma cruzi* complement regulatory protein family. Infect. Immun..

[47] Norris K.A., Bradt B., Cooper N.R., So M. (1991). Characterization of a *Trypanosoma cruzi* C3 binding protein with functional and genetic similarities to the human complement regulatory protein, decay-accelerating factor. J. Immunol..

[48] Nardy A.F.F.R., Freire de Lima C.G., Pérez A.R., Morrot A. (2016). Role of *Trypanosoma cruzi* trans-sialidase on the escape from host immune surveillance. Front. Microbiol..

[49] García G.A., Joensen L.G., Búa J., Ainciart N., Perry S.J., Ruiz A.M. (2003). *Trypanosoma cruzi: Molecular* identification and characterization of new members of the Tc13 family. Description of the interaction between the Tc13 antigen from Tulahuén strain and the second extracellular loop of the β1-adrenergic receptor. Exp. Parasitol..

[50] Joensen L., Borda E., Kohout T., Perry S., García G., Sterin-Borda L. (2003). *Trypanosoma cruzi* antigen that interacts with the β1-adrenergic receptor and modifies myocardial contractile activity. Mol. Biochem. Parasitol..

[51] Díaz Lozano I.M., De Pablos L.M., Longhi S.A., Zago M.P., Schijman A.G., Osuna A. (2017). Immune complexes in chronic Chagas disease patients are formed by exovesicles from *Trypanosoma cruzi* carrying the conserved MASP N-terminal region. Sci. Rep..

[52] Cura C.I., Mejía-Jaramillo A.M., Duffy T., Burgos J.M., Rodriguero M., Cardinal M.V., Kjos S., Gurgel-Gonçalves R., Blanchet D., De Pablos L.M. (2010). *Trypanosoma cruzi* I genotypes in different geographic regions and transmission cycles based on a microsatellite motif of the intergenic spacer of spliced leader genes. Int. J. Parasitol..

[53] Osuna A., Gamarro F., Castanys S., Ruiz Perez L.M. (1986). Inhibition of lysosomal fusion by *Trypanosoma cruzi* in peritoneal macrophages. Int. J. Parasitol..

[54] Campelo R., Díaz Lozano I., Figarella K., Osuna A., Ramírez J.L. (2015). *Leishmania major* telomerase TERT protein has a nuclear/mitochondrial eclipsed distribution that is affected by oxidative stress. Infect. Immun..

[55] Moreno M.L., Escobar J., Finamor I., Martinez-Ruiz A., Sastre J. (2014). Disulfide stress: A novel type of oxidative stress in acute pancreatitis. Free Radic. Biol..

[56] Shevchenko A., Jensen O.N., Podtelejnikov A.V., Sagliocco F., Wilm M., Vorm O., Mortensen P., Shevchenko A., Boucherie H., Mann M. (1996). Linking genome and proteome by mass spectrometry: Large-scale identification of yeast proteins from two dimensional gels. Proc. Natl. Acad. Sci. USA.

[57] Queiroz R.M., Ricart C.A., Machado M.O., Bastos I.M., de Santana J.M., de Sousa M.V., Roepstorff P., Charneau S. (2016). Insight into the exoproteome of the tissue-derived trypomastigote form of *Trypanosoma cruzi*. Front. Chem..

[58] De Pablos L.M., González G.G., Solano Parada J., Seco Hidalgo V., Díaz Lozano I.M., Gómez Samblás M.M., Cruz Bustos T., Osuna A. (2011). Differential expression and characterization of a member of the mucin-associated surface protein family secreted by *Trypanosoma cruzi*. Infect. Immun..

[59] Parisse P., Rago I., Ulloa Severino L., Perissinotto F., Ambrosetti E., Paoletti P., Ricci M., Beltrami A.P., Cesselli D., Casalis L. (2017). Atomic force microscopy analysis of extracellular vesicles. Eur. Biophys. J..

