# Supplementary material for: Biophysical and Biochemical Comparison of Extracellular Vesicles Produced by Infective and Non-Infective Stages of Trypanosoma cruzi
Source: Int J Mol Sci. 2021 May 13;22(10):5183. doi: 10.3390/ijms22105183 (PMC8153575; doi:10.3390/ijms22105183)
Supplement: Supplementary file 1 [file ijms-22-05183-s001.zip › ijms-1203006-supplementary.pdf]

Supplementary Materials

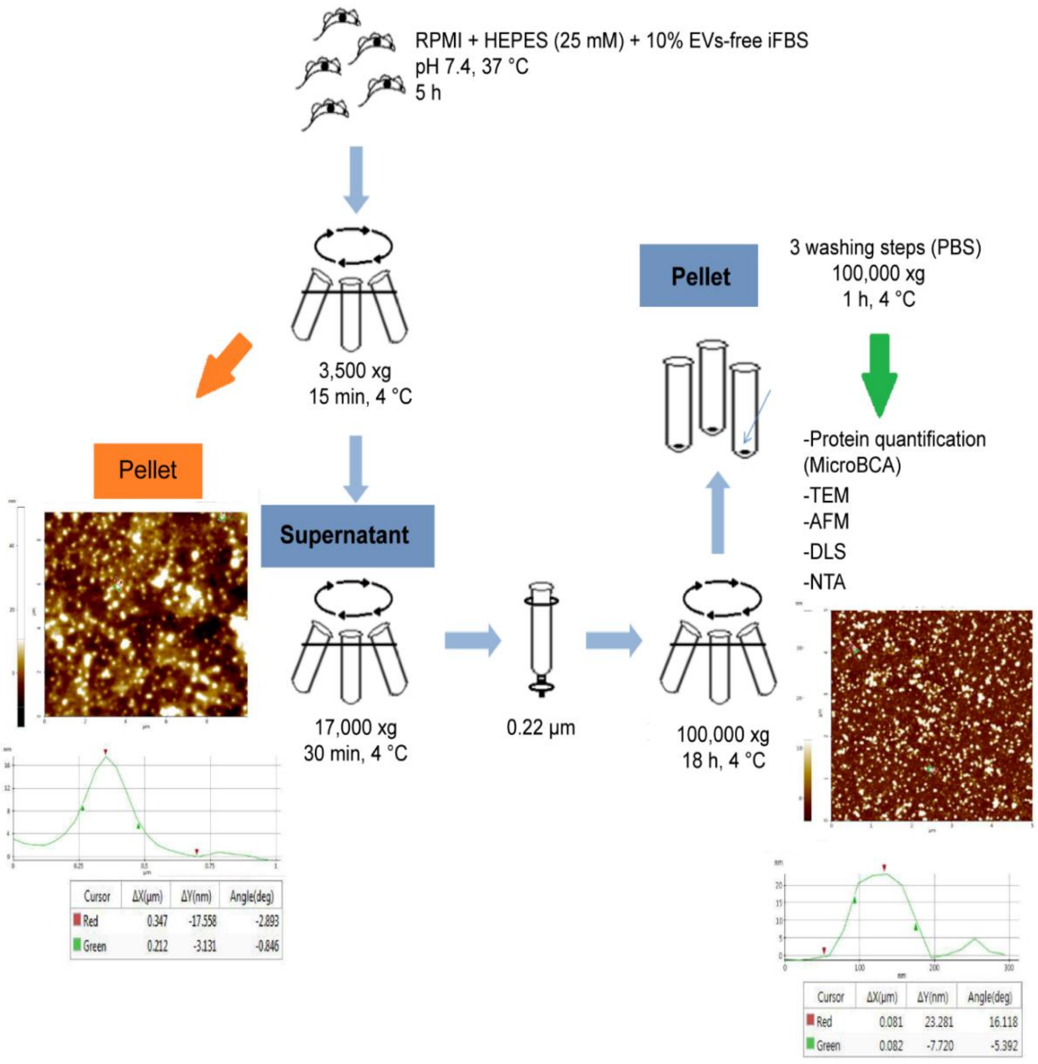

Supplementary Figure 1. Schematic representation of the isolation and purification of EVs of *T. cruzi* Pan4 strain.

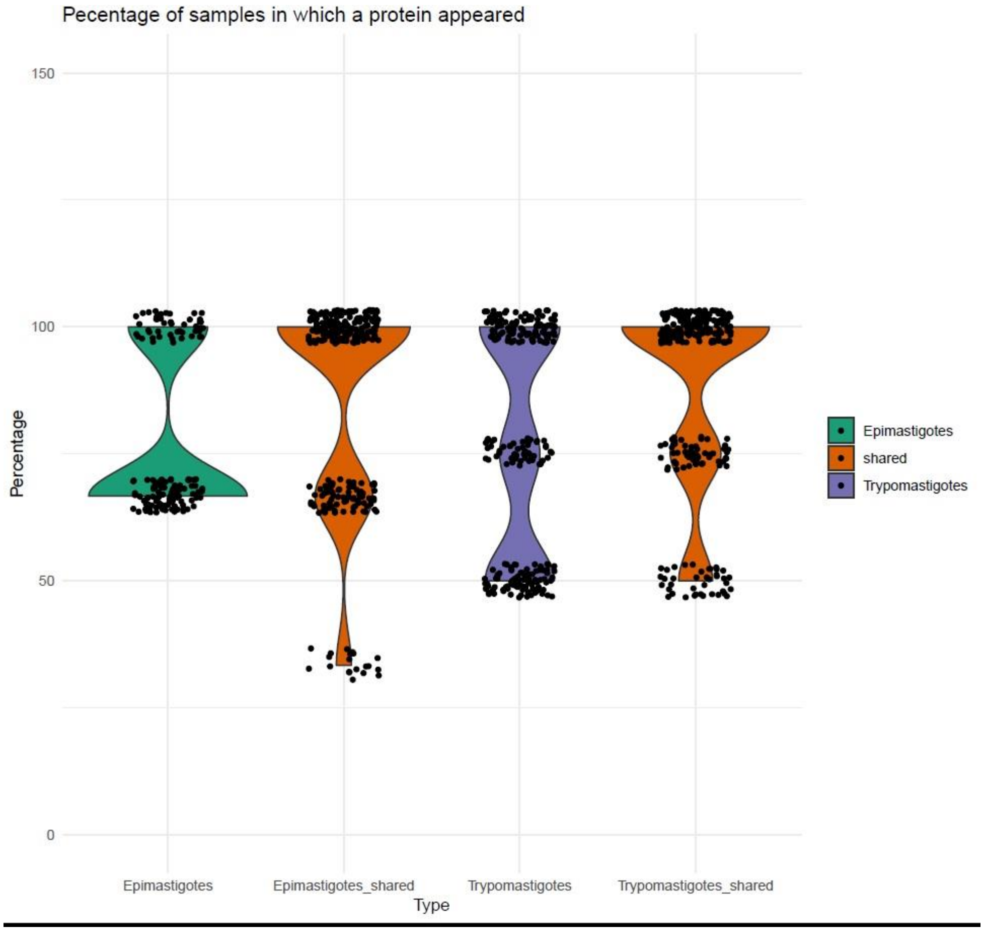

Supplementary Figure 2. Protein richness and frequency in EVs of *T. cruzi*. Violin plot showing the probability density (percentage, y-axis) of proteins in EVs of E, EVs of TCT and proteins present in both types of samples (shared).

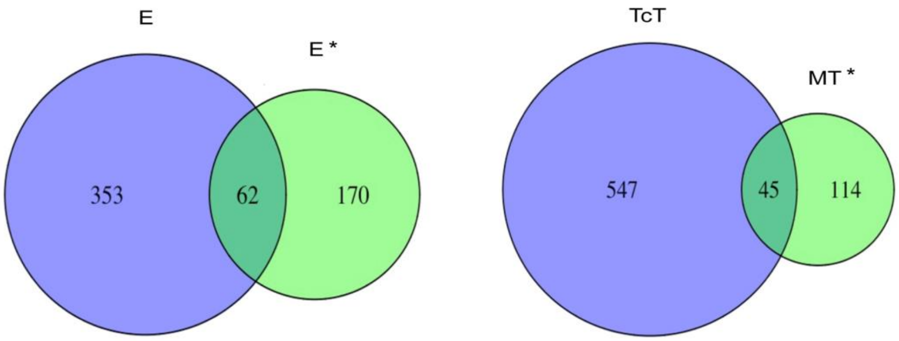

Supplementary Figure 3. Venn diagram for the comparison of the cargo of proteins of EVs of E and EVs of TCT of *T. cruzi* Pan4 strain employed in this study and the cargo of proteins of the EVs of E and MT stages isolated by Bayer Santos et al in 2013 (*) [24].

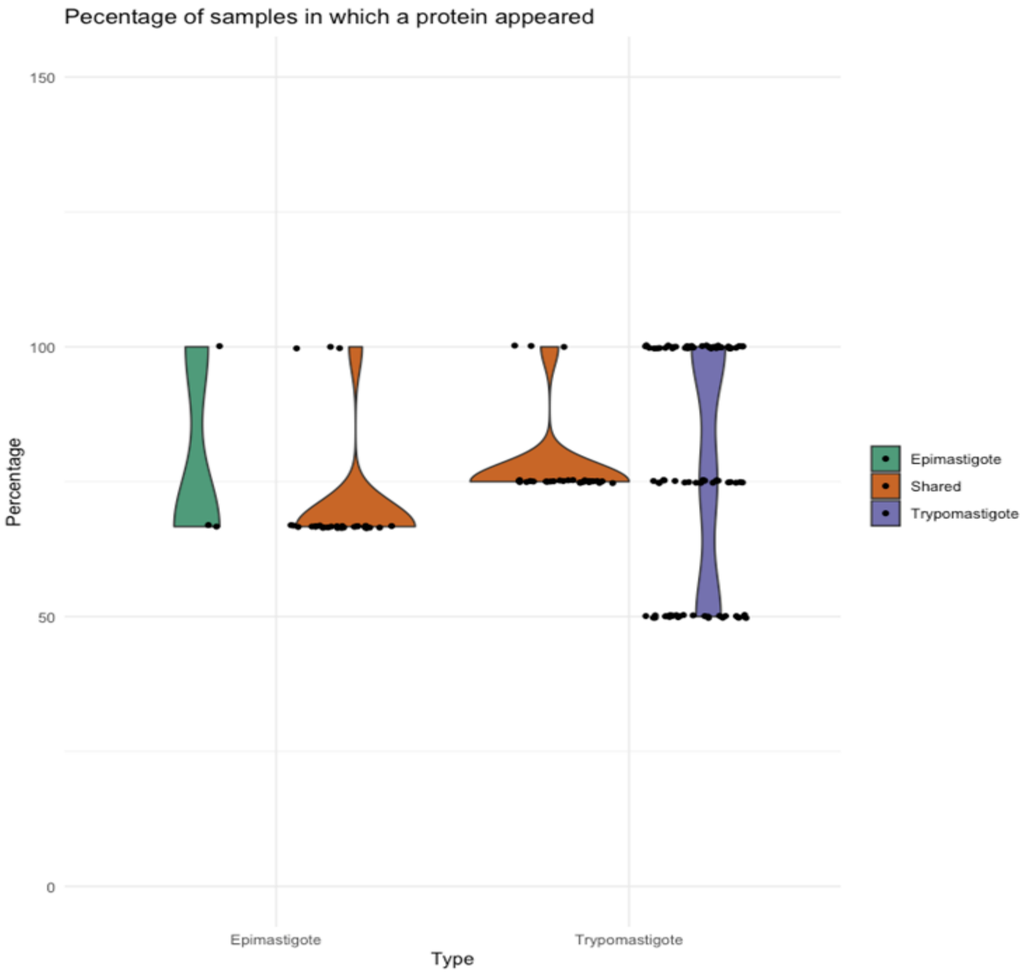

Supplementary Figure S4. Percentage of appearance of TS in EVs of *T. cruzi*: TS protein richness and appearance frequency. Violin plot showing the probability density (percentage, y-axis) of proteins in EVs of E, EVs of TCT and proteins present in both types of samples (shared).

Supplementary Methods

AFM Principle

The probe used in AFM is a cantilever (Si_3N_4) with a very sharp tip to scan the sample´s surface. The interaction between the tip and surface is the reference for scans. When the tip approaches the surface, the attractive force between the surface and the tip causes the cantilever`s deflection. However, if the cantilever is brought closer to the surface, such that the tip makes contact with it, repulsive forces increase and cause the cantilever to deflect away from the surface.

A laser beam is focused on the cantilever in such way that cantilever`s deflections are registered when the laser is moved from the middle position of the sensitive photodiode (PSPD). When the AFM tip sweeps the sample`s surface , it finds peaks and valleys that generate changes in the cantilever`s deflection and those movements are recorded in the PSPD.

AFM generates topographic maps of surface features using a feedback system that controls the height of the tip above the surface pixel by pixel and with subnanometric precision.

Structural and mechanical characterization of EVs

The structural and mechanical characterization using AFM has been reported based on the study of F-D curves as an output signal to extract quantitative results for several parameters such as elastic modulus, stiffness and adhesion according to Figure 6. F-D curves were sampled at 50 kHz. The elastic modulus is the ability of a material to resist being deformed elastically (returning to its original length) when stress (force) is applied and it is defined as the slope of its stress-strain curve (F-D curve), in the elastic deformation region. Stiffness is the measure of resistance to deformation in response to an applied force and adhesion is the tendency of surfaces to cling to one another.

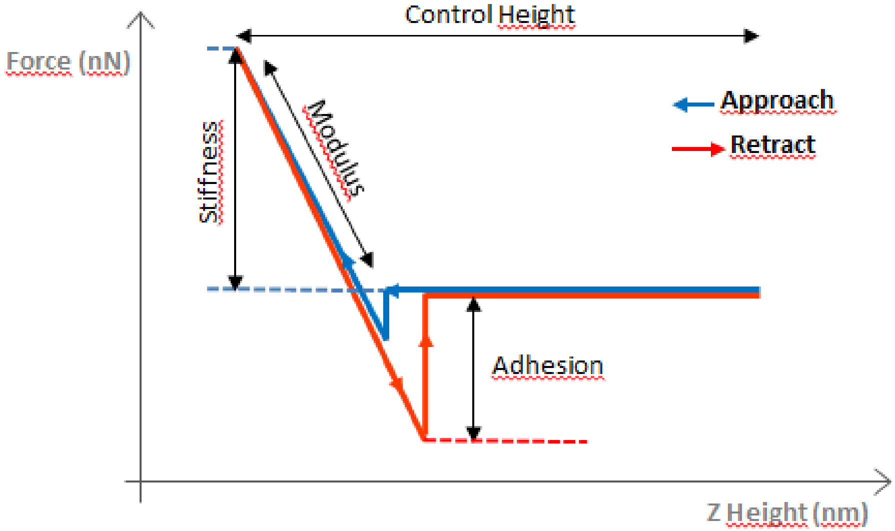

Supplementary Figure S5. F-D curve and parameters for nanomechanical properties.

Original blots and gels

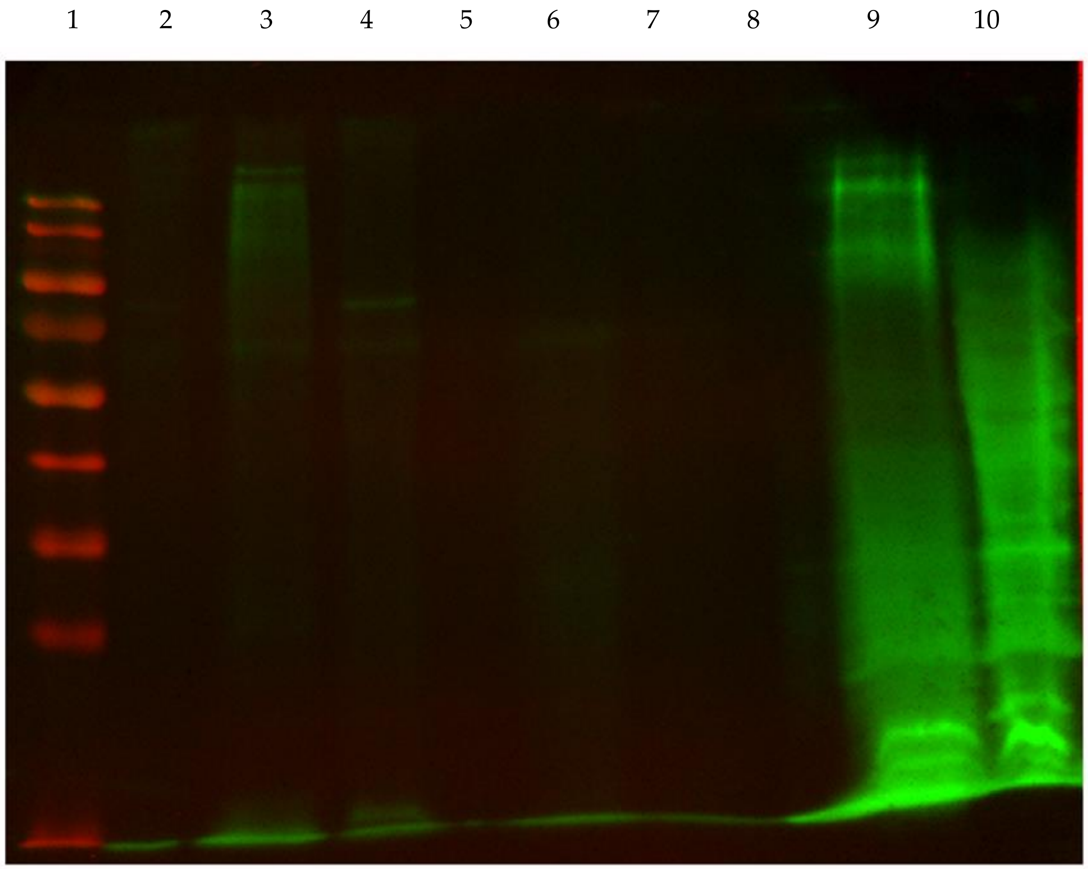

Supplementary Figure S6. Western blot and differential protein profile of E and TCT using polyclonal anti-*T. cruzi* polyclonal antibodies. Lane 1: molecular weight marker; lane 9: whole lysates of TCT; lane 10: wholelysates of E.

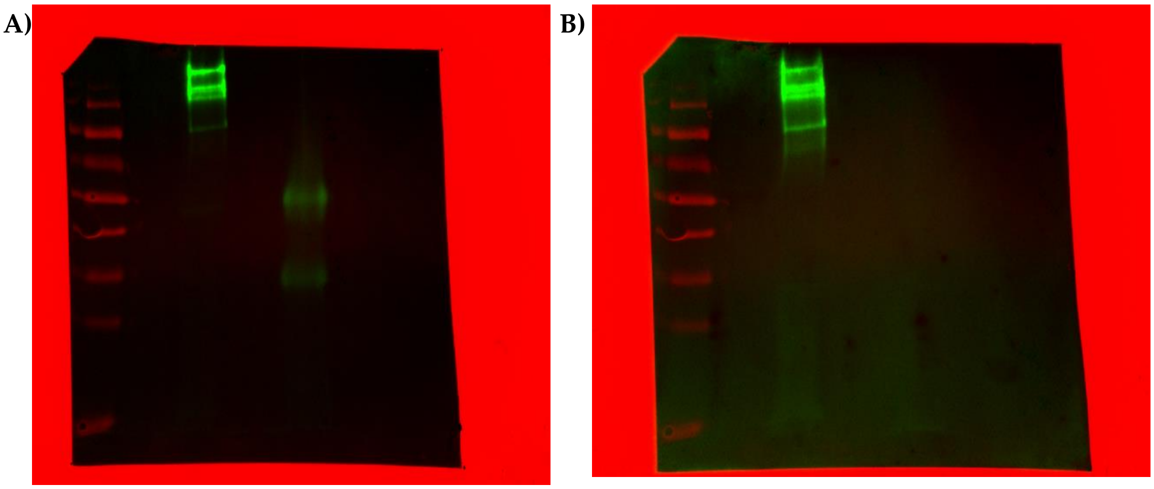

Supplementary Figure S7. *Trans*-sialidase (mAb 39) in whole lysates of TCT (A, lane) and *trans*-sialidase (mAb 39) in EVs of TCT (B, lane 3).

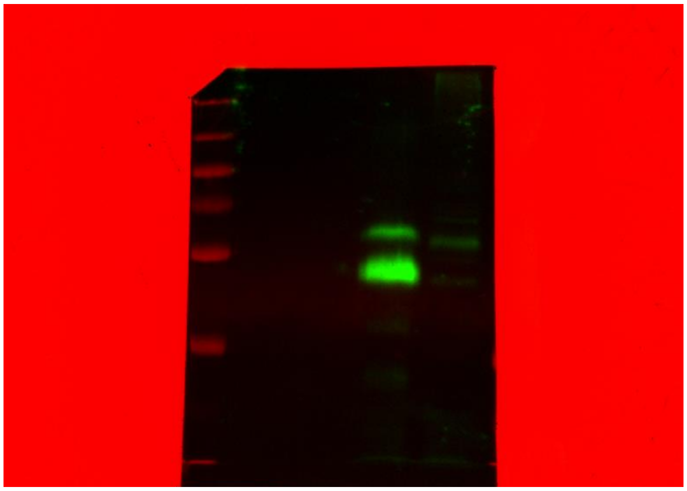

Supplementary Figure S8. Cruzipain in whole lysates of TCT (lane 4) and cruzipain in EVs of TCT (lane 5).

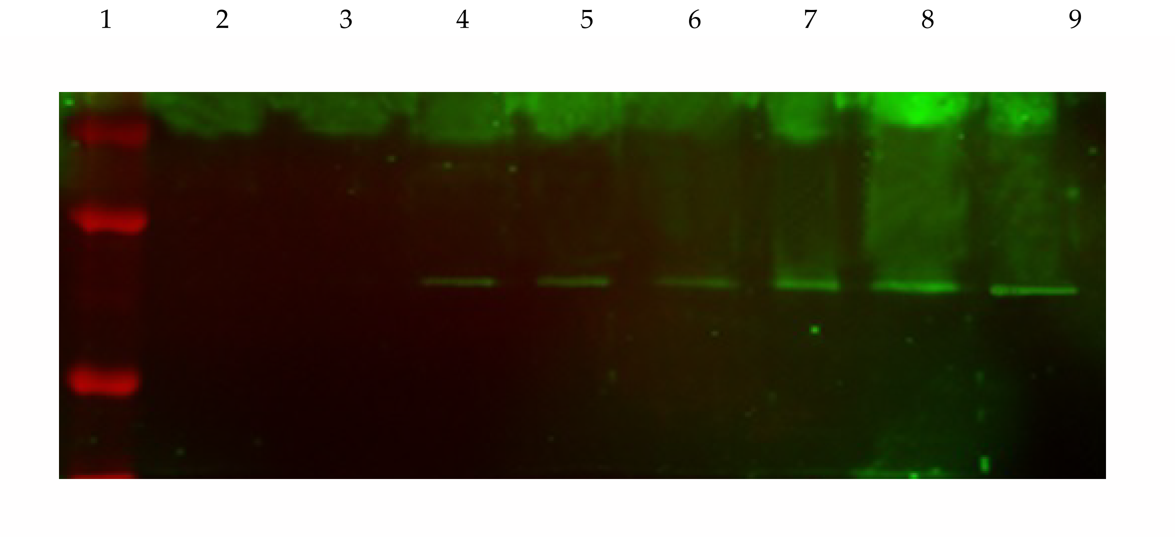

Supplementary Figure S9. PHB2 in EVs of E (lanes 4 and 5) and PHB2 in whole lysates of E (Lines 6-9).

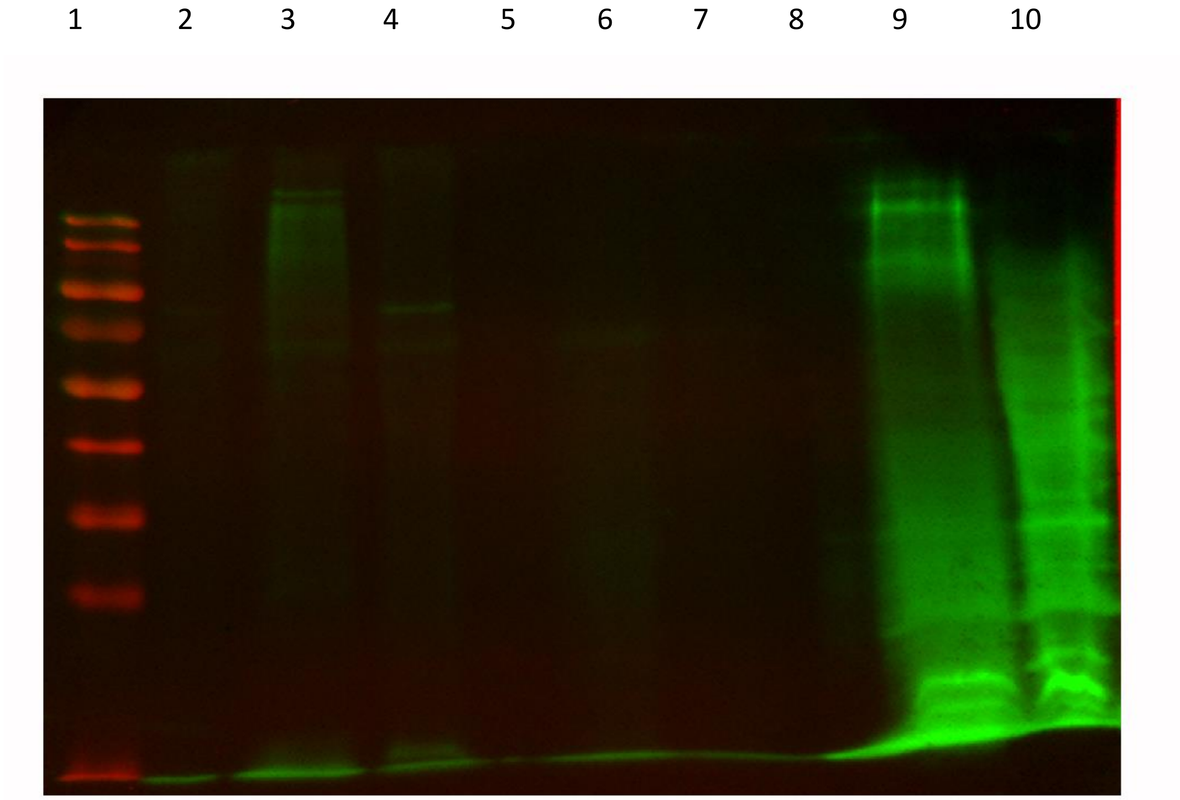

Supplementary Figure S10. Western blot of EVs of E and EVs of TCT treated/untreated with proteinase K and incubated with anti-TS antibodies. Lane 1: molecular weight marker; lane 2: treated EVs of TCT; lane 3: untreated EVs of TCT; lane 4: untreated EVs of E; lane 5: treated EVs of E; lane 9: whole lysates of TCT; Lane 10: wholelysates of E.
